# Prognostic potential of ERCC1 protein expression and clinicopathologic factors in stage III/N_2_ non-small cell lung cancer

**DOI:** 10.1186/1749-8090-8-149

**Published:** 2013-06-10

**Authors:** Dong Yan, Ping Wei, Guangyu An, Wenming Chen

**Affiliations:** 1 Department of Oncology, Beijing Chao-Yang Hospital affiliated to Capital Medical University, Workers Stadium South Road, Beijing, China; 2 Department of Pathology, Beijing Chao-Yang Hospital affiliated to Capital Medical University, Workers Stadium South Road, Beijing, China; 3 Department of Hematology, Beijing Chao-Yang Hospital affiliated to Capital Medical University, Workers Stadium South Road, Beijing, China

**Keywords:** Excision repair cross complementation 1, Non-small lung cancer, Number of involved lymph nodes, Prognosis, Skip metastasis

## Abstract

**Background:**

Pathological stage III/N_2_ non-small cell lung cancer (NSCLC) is heterogeneous, and the optimal prognostic marker for survival remains unclear in Chinese patients. The aim of the present study was to assess the prognostic value of the clinicopathologic features and excision repair cross-complementing group-1 (ERCC1) in resected p-stage III/N_2_ NSCLC patients that received cisplatin-based adjuvant chemotherapy.

**Methods:**

Clinical data concerning 115 patients with histopathologically confirmed stage III/N_2_ NSCLC who underwent a complete resection were reviewed retrospectively. All patients received cisplatin-based adjuvant chemotherapy. The protein expression levels for ERCC1 were immunohistochemically examined in 115 patients. The relationship between the ERCC1 protein expression level and the clinical outcomes of the patients was then observed.

**Results:**

The 5-year survival rate and median survival time of patients with pathological stage III/N_2_ NSCLC after surgery and postoperative chemotherapy was 27.0% and 28.0 months, respectively. Survival of patients with ERCC1 negative tumors was significantly longer than those with ERCC1 positive tumors (p = 0.004). However, it was not entirely clear whether adjuvant chemotherapy with cisplatin-based agents was beneficial for ERCC1-negative patients with p-stage III/N_2_. A multivariate analysis of survival in patients with stage III/N_2_ NSCLC showed that surgical procedure (pneumonectomy vs. lobectomy; p = 0.001), number of involved lymph nodes (≤5 vs. >5; p = 0.001) and ERCC1 protein expression (negative vs. positive; p = 0.012) were significant prognostic factors. In addition, the prognosis of patients with skip mediastinal lymph node metastasis showed a tendency for improved survival, but this was no significant (p = 0.432).

**Conclusions:**

Findings from this retrospective study suggested that the number of involved lymph nodes and the type of pulmonary resection are significant and independent prognosis factors in patients with p-stage III/N_2_ NSCLC. In addition, it was found that ERCC1 protein expression might play an important role in the prognosis of p-stage III/N_2_ NSCLC patients treated with cisplatin-based adjuvant chemotherapy.

## Background

Because of its aggressiveness, lung cancer is often in an advanced stage by the time it is diagnosed. The range in the survival of patients with pathological stage III/N_2_ locally advanced NSCLC associated with various prognostic factors suggests that they are a heterogeneous group [1,2]. Many studies have evaluated the validity of various prognostic factors among p-N_2_ NSCLC patients in order to establish a treatment strategy [3-5]. However, some reports conflict, and in general the associations do not appear sufficiently strong to be of value in formulating a clinical treatment plan. Recent studies have investigated a number of tumor biomarkers for prognostic and predictive utility when considering systemic therapy and the most prominent amongst these is the excision repair cross-complementation group 1 (ERCC1) protein. ERCC1 is one of the key enzymes of the nucleotide excision repair pathway, which is essential for the removal of platinum-DNA adducts. Recent studies have demonstrated that the expression levels of ERCC1 are related to a survival benefit from cisplatin-based chemotherapy among patients with advanced NSCLC [6-8]. In an adjuvant setting, patients with ERCC1-negative tumors exhibited prolonged survival relative to those with ERCC1-postive tumors. These findings suggest that ERCC1 expression is a double-edged sword in NSCLC, simultaneously being a significant and independent prognostic factor of survival in NSCLC and also being associated with increased resistance to platinum-based chemotherapy [9-14].

In the present study, we retrospectively investigated whether the clinicopathologic factors and the expression levels of ERCC1 in tumor tissue, measured using immunohistochemical staining, were associated with prognosis in Chinese patients who underwent surgery for p-stage III-N_2_ NSCLC.

## Methods

### Patient selection

Patients with histopathologically confirmed stage III/N_2_ NSCLC who underwent a complete resection, either by means of lobectomy or pneumonectomy, and were administered with postoperative systemic chemotherapy were evaluated retrospectively between January 2005 and December 2009 at Beijing Chao-Yang Hospital, China. Mediastinal lymphadenectomy was routinely performed. The postoperative chemotherapy regimen was cisplatin at a dose of 75 mg/m^2^ on day 1 plus gemcitabine at a dose of 1250 mg/m^2^ on days 1 and 8 every 3 weeks (GP), or cisplatin at a dose of 75 mg/m^2^ on day 1 plus paclitaxel at a dose of 175 mg/m^2^ on day 1 every 3 weeks (TP) [15]. The patients who had received preoperative therapy were excluded.

Resected specimens were sent to our pathology department for histopathological and immunohistochemical examination. The histopathological findings were classified according to the World Health Organization criteria, and the AJCC TNM staging system (7th edition) was also employed [16,17]. Skip metastasis is lymph node metastasis that skips lymph node stations that are in close proximity and occurs at a considerable distance from the primary tumor. Lymph node metastases from lung cancer may skip the intralobar lymph nodes and move directly to the mediastinum. In our study, skip mediastinal lymph node metastasis was defined as mediastinal lymph node metastasis (N_2_) without peribronchial and/or hilar lymph node metastasis (N_1_). The preoperative assessments included chest roentgenography and computed tomography (CT) of the chest and upper abdomen. Clinical N_2_ status was defined by the presence of a lymph node measuring more than 1 cm in short axis diameter. Bone scintigraphy was performed to detect bone metastasis. Magnetic resonance imaging of the brain was routinely employed for the assessment of distant metastasis. Bronchoscopy was routinely performed to obtain a pathological diagnosis using transbronchial lung biopsy, and to evaluate endobronchial staging.

Follow-up information was obtained from all patients through office visits or telephone interviews, either with the patient or with a relative. The patients were evaluated every 3 months using chest roentgenography, and chest CT scans were performed every 6 months for the first 2 years after surgery and annually thereafter. The survival period was calculated using the day of lung resection as the first day, and the day of death or the last follow-up as the last day. The patients’ records including their clinical data, preoperative examination results, details of any surgeries, histopathological findings, and the TNM stages of all patients were also reviewed.

### Immunohistochemical staining

A total of 115 human stage III/N_2_ lung cancer specimens were obtained. The patients were fully informed and gave their consent for collection of clinical samples. The primary tumor was resected and specimens were fixed in 10% formalin and embedded in paraffin. The longest diameter of the tumors was measured, and one or two 5-μm thick sections were obtained for each centimeter of the tumor from the areas containing viable tumor tissue at a maximum ratio with no (or minimum) hemorrhages or necrosis. Antigen retrieval was performed by adding citrate buffer (pH 6.0) and heating in a microwave oven (20 minutes at 100°C). All of the procedures were performed in accordance with the antibody manufacturers’ protocols. The primary tumor sections were incubated with anti-ERCC1 primary antibodies at a 1:100 dilution (8F1: Thermo-Lab Vision). The reaction was visualized using a 3, 3-diaminbenzidine (DAB: Roche) substrate system. Then contrast staining was performed using Mayer’s hematoxylin.

### Evaluation of the ERCC1 expression level

ERCC1 staining was independently evaluated under a light microscope at a magnification of ×400 by two pathologists who were blinded to the clinical data. Tumor nuclear staining intensity was graded on a scale of 0–3. The percentage of positive tumor nuclei was evaluated, and a proportion score was attributed (0 if 0%; 0.1 if 1%–9%; 0.5 if 10%–49%; and 1.0 if ≥50%), as previously described [10]. The intensity and proportion scores were then multiplied to give the semiquantitative H-score. The median value of all of the H scores was chosen a priori as the cutoff point for separating ERCC1-positive tumors from ERCC1-negative tumors.

### Statistical analysis

Cases were evaluated for demographic, surgical, and pathological variables, and the distributions of these variables were compared using the *χ*^2^ test or Fisher’s exact test. Patient survival was analyzed by means of the Kaplan–Meier method, using time zero as the date of thoracotomy and death as the endpoint. Differences in survival were determined using the log-rank test for univariate analysis, and prognostic factors were included in a multivariate analysis using the Cox proportional hazards regression model. Results were considered significant at p < 0.05.

## Results

**Figure 1:**
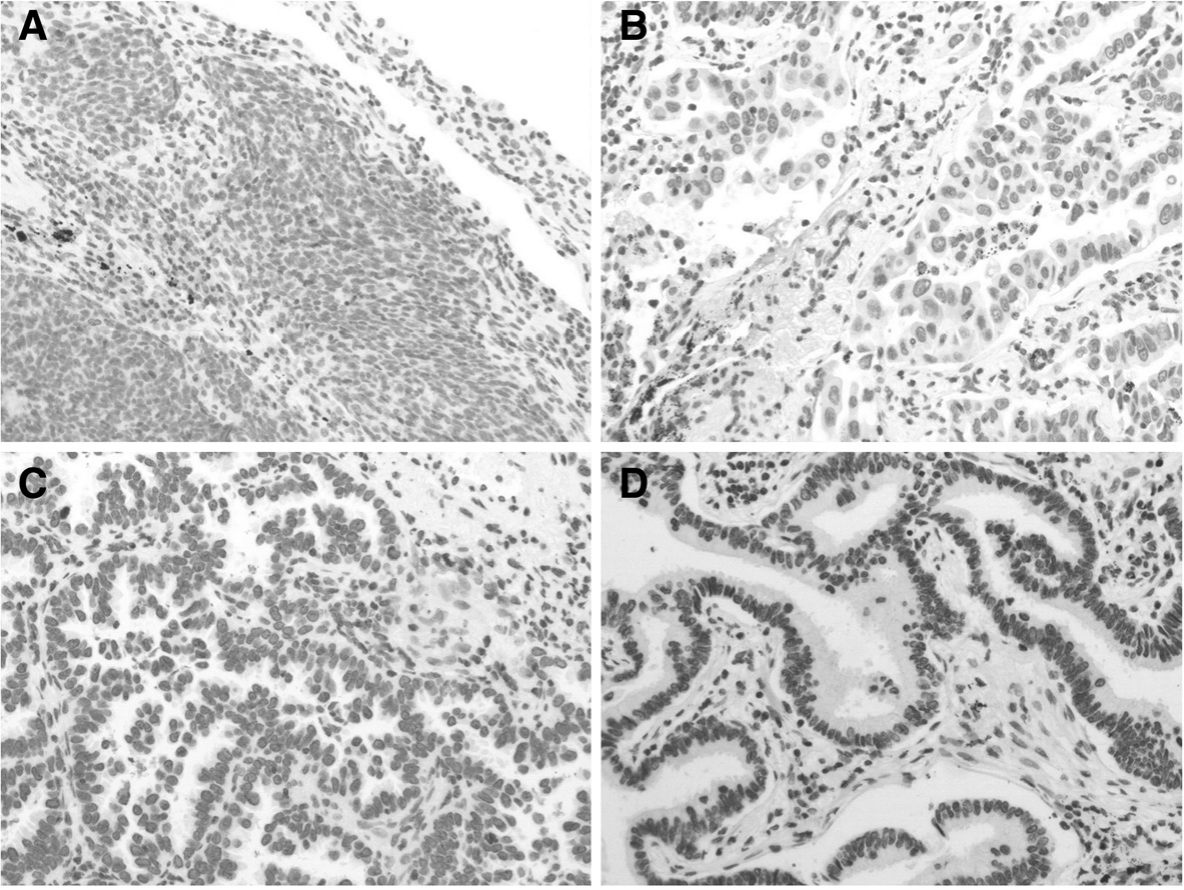
**Examples of ERCC1-expression scores obtained using immunohistochemical analysis.** Score 0 (**A**), 1 (**B**), 2 (**C**) and 3 (**D**) correspond to none, weak, moderate, and strong, respectively.

A total of 115 patients met the enrollment criteria and were entered into the study from January 2005 to December 2009 at the Beijing Chao-Yang Hospital, China. The patients included 81 males and 34 females. The median age of all patients was 62 years (ranging from 33–80 years). Seventy four patients (64.3%) had a smoking habit. The histological types included 78 adenocarcinomas (67.8%), 30 squamous cell carcinomas (26.1%) and 7 large cell carcinomas (6.1%). There were 29 patients graded as T_1_, 54 as T_2_, 22 as T_3_, and 10 as T_4_ (stage IIIB). A pneumonectomy was performed in 25 (21.7%), and a lobectomy in 90 (78.3%). The average number of dissected lymph nodes (N_1_ and N_2_) was 20.0 (range: 5–42). The average number of involved lymph nodes (N_1_ and N_2_) was 5.0 (range: 1–24). Skip mediastinal lymph node metastasis (N_1_ negative) was demonstrated in 34 patients (29.6%), and mediastinal lymph node metastasis with N_1_ disease (N_1_ positive) was found in 81 patients. During this study, 76 patients received the GP chemotherapy regimen, and 39 patients received the TP chemotherapy regimen for 4 to 6 cycles, respectively. Patient characteristics are presented in Table 1. ERCC1-positive expression (H score ≥1.0) was observed in 71 of the 115 tumors examined using immunohistochemical staining. Examples of the immunohistochemical staining patterns are show in Figure 1. The relationship between the immunohistochemical staining patterns and the clinicopathological factors were examined (Table 1). No significant associations were found between the ERCC1 expression status and age, sex, smoking, pathological stage, operative procedure, or the number of involved lymph nodes.

**Table 1:** Clinicopathological factors and ERCC1 protein expression

**Characteristics**	**No. of cases (%) (n = 115)**	**ERCC1 (+) (%)**	**ERCC1 (−) (%)**	**p-Value**
Age				
≤65 years	69 (60.0%)	42 (60.9%)	27 (39.1%)	0.814
>65 years	46 (40.0%)	29 (63.0%)	17 (37.0%)	
Gender				
Female	34 (29.6%)	17 (50.0%)	17 (50.0%)	0.093
Male	81 (70.4%)	54 (66.7%)	27 (33.3%)	
Smoking				
Ever	74 (64.3%)	44 (59.5%)	30 (40.5%)	0.499
Never	41 (35.7%)	27 (65.9%)	14 (34.1%)	
Histology type				
Adenocarcinoma	78 (67.8%)	52 (66.7%)	26 (33.3%)	0.114
Others	37 (32.2%)	19 (51.4%)	18 (48.6%)	
Pathological-stage				
IIIA	105 (91.3%)	67 (63.8%)	38 (36.2%)	0.178
IIIB	10 (8.7%)	4 (40.0%)	6 (60.0%)	
T factor				
T1、T2	83 (72.2%)	48 (57.8%)	35 (42.2%)	0.165
T3、T4	32 (27.8%)	23 (71.9%)	9 (28.1%)	
Operative procedure				
Pneumonectomy	25 (21.7%)	15 (60.0%)	10 (40.0%)	0.840
Lobectomy	90 (78.3%)	56 (62.2%)	34 (37.8%)	
Number of involved lymph nodes (N_1_+N_2_)				
≤5	59 (51.3%)	32 (54.2%)	27 (45.8%)	0.089
>5	56 (48.7%)	39 (69.6%)	17 (30.4%)	

**Figure 2:**
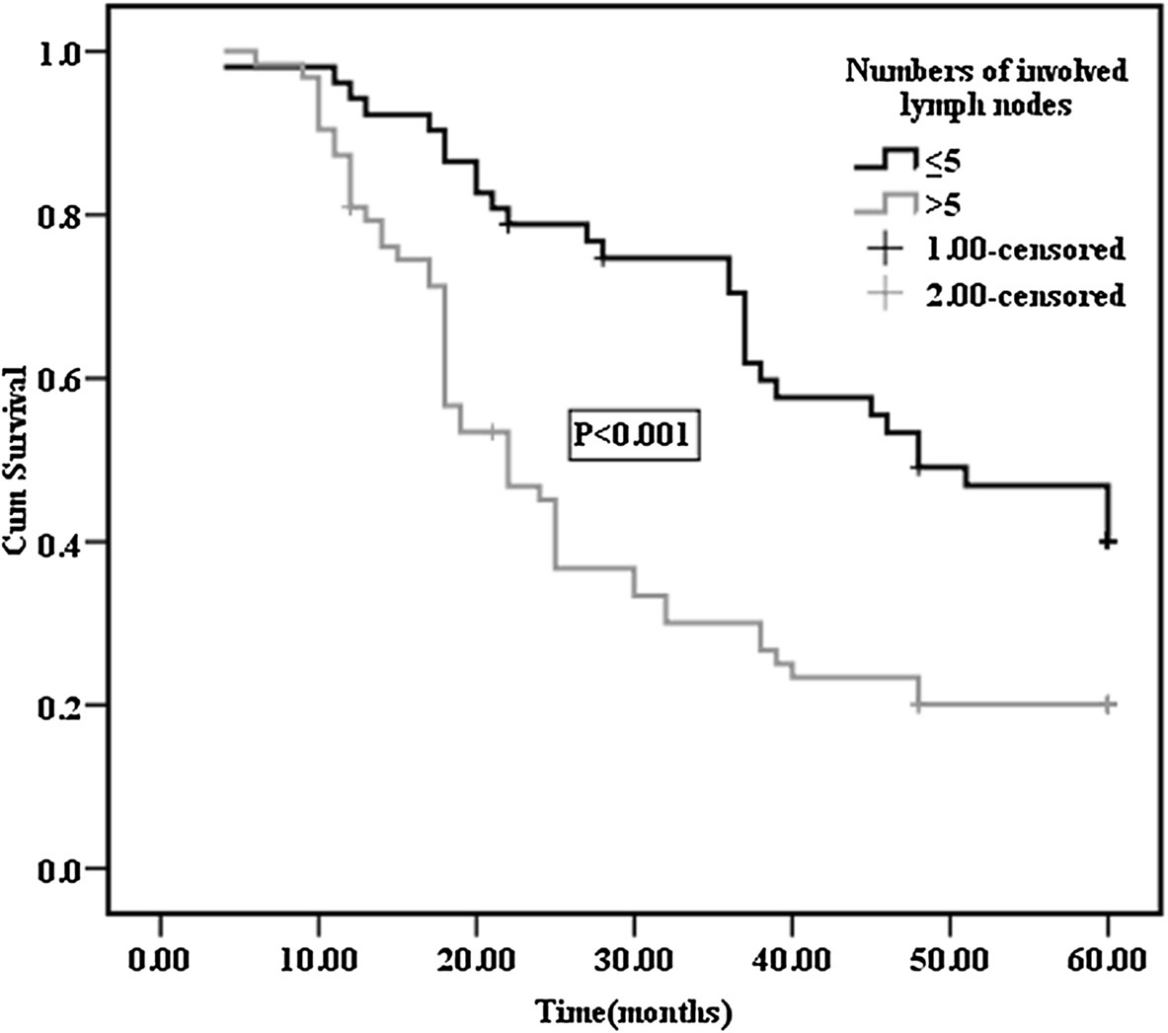
**Overall survival curves after surgery in p-N**_**2 **_**patients according to the numbers of involved lymph nodes.** The 5-year survival rates of patients with ≤5 positive lymph nodes and >5 positive lymph nodes were 40.2% and 20.0%, respectively.

**Figure 3:**
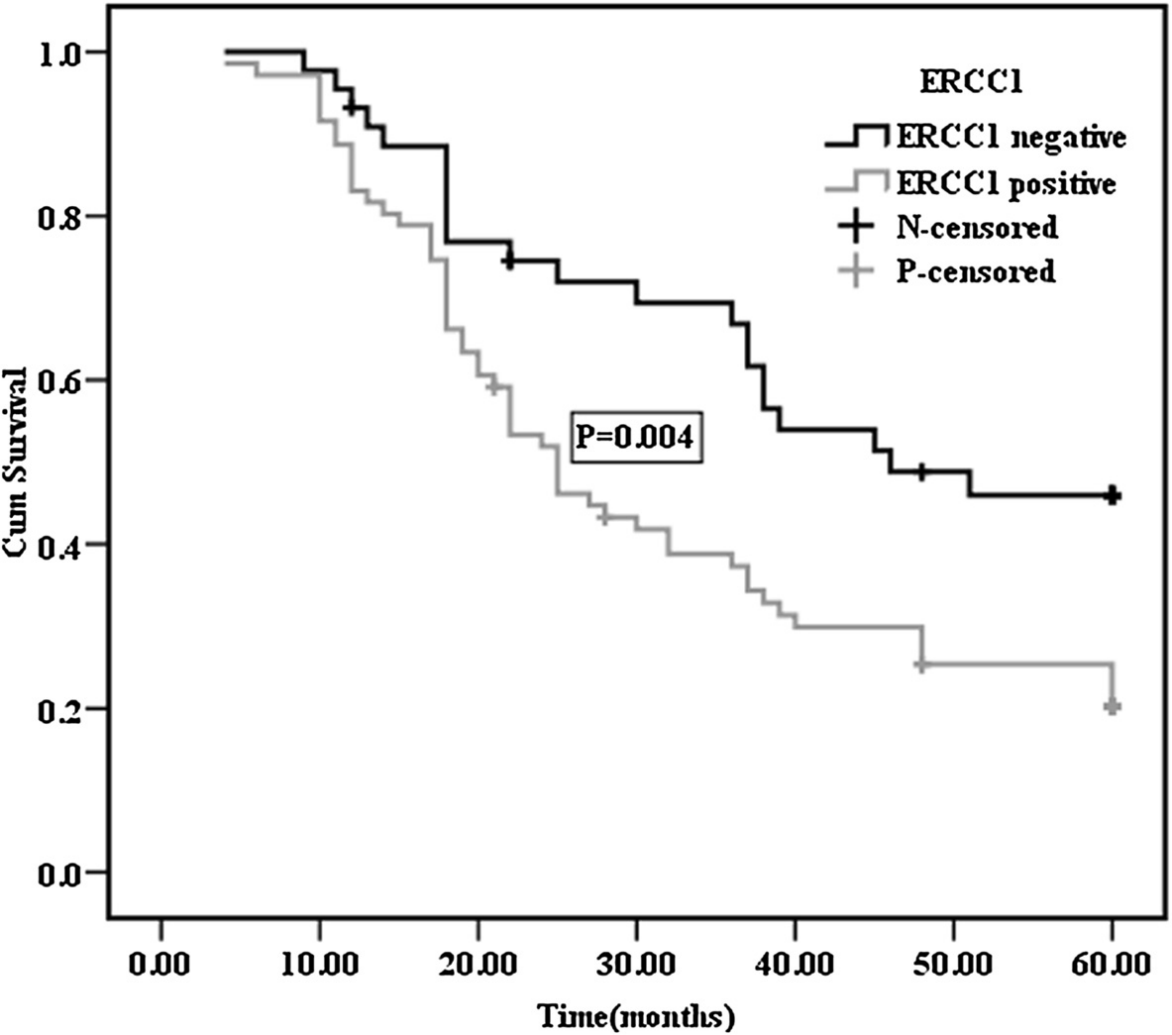
**Survival curves for NSCLC patients with negative or positive expression of ERCC1.** The 5-year survival rate was 46% for patients with ERCC1 negative expression and 20.3% for patients with ERCC1 positive expression.

The median duration of observation was 34 (4–68) months. The 5-year survival rate and median survival time after surgery of patients with pathological stage III/N_2_ NSCLC was 27.0% and 28.0 months (95% CI, 18.0-60.0), respectively. The 5-year survival rate of patients with mediastinal lymph node metastasis (N_2_) that had skip mediastinal lymph node metastasis (without N_1_ lymph node metastasis) was 32.8%; however, the 5-year survival rate of patients with mediastinal lymph nodes metastasis (N_2_) with N_1_ disease was 28.5%. The prognosis of patients with skip mediastinal lymph node metastasis showed a tendency for improved survival, but this was not significant (p = 0.432). With regard to the total number of involved lymph nodes, the 5-year survival rate of patients with 5 or less than 5 positive lymph nodes was 40.2%; it was 20.0% for patients with more than 5 positive lymph nodes (Figure 2). Differences between the two groups were statistically significant (p < 0.001). The effects of the type of lung resection were assessed, and the 5-year survival rate was found to be 33.6% for patients who underwent lobectomy, and 16% for patients who underwent pneumonectomy (p = 0.001). In addition, significant differences were observed in the survival rates of patients with T_1_/T_2_ relative to T_3_/T_4_ disease (p = 0.003). Age, gender and histopathological type did not affect survival (Table 2). The 5-year survival rates of patients was 46% for ERCC1 negative expression and 20.3% for ERCC1 positive expression; this difference was statistically significant (p = 0.004; Figure 3). Multivariate analysis of survival in patients with stage III/N_2_ NSCLC showed that surgical procedure (pneumonectomy vs. lobectomy; p = 0.001), number of involved lymph nodes (≤5 vs. >5; p = 0.001) and ERCC1 protein expression (negative vs. positive; p = 0.012) were significant prognostic factors (Table 3). Age, T factor, gender, histology, and skip metastasis did not affect survival.

**Table 2:** Evaluation of patient survival using univariate analysis (log-rank test)

**Characteristics**	**n**	**5-Year survival (%)**	**p-Value**
Age			
≤65 years	69	27.2	0.981
>65 years	46	34.4	
Gender			
Female	34	33.9	0.690
Male	81	28.0	
Histology type			
Adenocarcinoma	78	30.5	0.894
Others	37	28.1	
T factor			
T1、T2	83	37.7	0.003*
T3、T4	32	15.4	
Operative procedure			
Pneumonectomy	25	16.0	0.001*
Lobectomy	90	33.6	
Number of involved lymph nodes (N_1_+ N_2_)			
≤5	59	40.2	<0.001*
>5	56	20.0	
Skip metastasis			
Yes	34	32.8	0.432
No	81	28.5	
ERCC1 protein expression			
negative	44	46.0	0.004*
positive	71	20.3	

**Table 3:** The results of the multivariate analysis of overall survival

**Variables**	**Overall survival**	**p-Value**
	**Hazard ratio (95% CI)**	
Age		
≤65 years	0.938(0.564–1.561)	0.805
>65 years	1	
Gender		
Female	0.684(0.387–1.208)	0.191
Male	1	
T factor		
T1、T2	0.805(0.460–1.408)	0.447
T3、T4	1	
Histology		
Others	1.070(0.630–1.817)	0.802
Adenocarcinoma	1	
Operative procedure		
Pneumonectomy	2.455(1.467–4.108)	0.001*
Lobectomy	1	
Skip metastasis		
Yes	0.834(0.503–1.384)	0.483
No	1	
Number of involved lymph nodes		
≤5	0.449(0.278–0.724)	0.001*
>5	1	
ERCC1 protein expression		
negative	0.526(0.318–0.870)	0.012*
positive	1	

## Discussion

NSCLC represents one of the most common and aggressive solid tumors, and it is difficult to cure. The Japanese Lung Cancer Registry Study of 6644 resected NSCLC cases in Japan reported that the 5-year survival rates of patients with stage IIIA and IIIB were 29.8% and 19.3%, respectively [18]. The prognosis of N_2_ NSCLC patients has remained poor, and most oncologists believed that surgery alone is not sufficient to cure patients with N_2_ disease. During the past decade, most patients with N_2_ have received perioperative chemotherapy to improve their survival. However, patients with pN_2_ NSCLC are a heterogeneous population, and optimal therapeutic selection for stage III NSCLC is controversial. The 5-year survival rate for patients with p-stage III/N_2_ was 27% in the current series, which is consistent with the Japanese Lung Cancer Registry Study. This suggested that some cases can be cured by surgery alone, while others should be considered for more intensive treatments in order to potentially achieve a cure. The valuable factors regarding multimodality therapy for the improvement of prognosis in stage III/N_2_ disease are unclear.

Some studies have shown that a single station of mediastinal-node metastasis was an acceptable prognostic predictor [19], and another study demonstrated that the highest lymph node involvement, skip metastasis and single station involvement were associated with prognosis [20-22]. Skip metastasis in our study was defined as the presence of mediastinal lymph node metastasis (N_2_ disease) without intralobar and/or hilar nodal involvement (N_1_). In our study, the patients with skip metastasis showed a tendency for improved survival; however, there was no significant survival difference between patients with skip metastasis relative to those without skip metastasis (p = 0.432).

The present study focused on the number of involved lymph nodes in regional lymph nodes (N_1_ and N_2_). The 5-year survival rate of the patients at stage III/N_2_ was decreased according to the total number of involved lymph nodes (N_1_+N_2_) present. The patients with ≤5 lymph node metastases had a significantly better prognosis than those with >5 lymph node metastases. In the multivariate analysis, the number of involved lymph node was also found to be a significant independent prognostic factor for patients with p-stage III/N_2_ NSCLC.

Surgical intervention still plays a crucial role in selected cases with p-stage III/N_2_ NSCLC. Lobectomy may be safely performed following induction therapy while pneumonectomy may carry a high and possibly unacceptable rate of perioperative mortality [23,24]. This retrospective study tried to clarify the prognostic importance of the type of pulmonary resection in patients with p-stage III/N_2_ NSCLC, who underwent complete dissection of the mediastinal lymph nodes. Multivariate analysis demonstrated that undergoing pneumonectomy was significantly associated with a shorter overall survival (OS) (hazard ratio [HR] 2.455; p = 0.001).

The survival outcomes of patients with p-stage III/N_2_ NSCLC have been poor when they were treated with surgery alone. Numerous studies have investigated induction chemotherapy, radiotherapy, and chemoradiotherapy with the objective of improving the outcome for this high-risk population. The present status of postoperative adjuvant chemotherapy for the treatment of completely resected p-stage III/N_2_ NSCLC is based on the results of large-scale phase III trials involving cisplatin-based regimens, such as IALT and ANITA studies, and a recent individual patient meta-analysis [25-27]. However, the appropriate application and sequence of these treatments is still the subject of ongoing studies. Thus, new biomarkers are required for use in treatment decision-making. Currently, the most frequently reported candidate marker is ERCC1, implicated in DNA repair.

ERCC1 is the limiting factor in nucleotide excision repair, which removes platinum-DNA adducts. ERCC1 may also be involved in the repair of DNA double-strand breaks, especially those induced by interstrand cross-links. It is possible that the presence of ERCC1 reflects an inherent biologic characteristic of the tumor. The ERCC1 expression level has recently been reported to be a prognostic factor in the survival of patients with early stage NSCLC [8,12]. Our study evaluated the effect of intratumoral ERCC1 expression on the survival of patients with completely resected p-stage III/N_2_ NSCLC. Results showed that the survival for patients with ERCC1 negative tumors was significantly longer than those for patients with ERCC1 positive tumors. This suggested that adjuvant chemotherapy with cisplatin-based agents may be beneficial for ERCC1-negative patients. Olaussen et al. [12] also reported that ERCC1 protein expression status was associated with favorable prognosis in patients treated with cisplatin-base adjuvant chemotherapy, similar to data reported in another study [28]. Furthermore, in the multivariate analysis, ERCC1 was identified as an independent predictor of survival. Further validation of these findings may help us to choose molecular-based personalized multimodality therapy.

The limitations of this study were: (I) it was retrospective and many factors could have influenced survival; (II) the sample size was limited, so the prognostic significant of skip metastasis with regard to OS was not confirmed; and (III) the ERCC1 positive expression rate in our study was higher than that reported in the IALT trial. Possible explanations might be that ours was a small sample size retrospective study, and all of the Chinese patients were diagnosed with p-stage III/N_2_ NSCLC.

## Conclusions

In conclusion, the survival of patients with p-stage III/N_2_ NSCLC depends on numerous factors, and accurately determining the prognosis of disease in these patients is unclear. Therefore, valuable biomarker fitting to determine the subgroup of patients who have a relatively poor prognosis in the Chinese population is needed. Our study suggested that the number of involved lymph nodes and the type of pulmonary resection are significant and independent prognostic factors in patients with p-stage III/N_2_ NSCLC. In addition, the current study revealed that DNA repair genes may play an important role in the prognosis of locally advanced NSCLC patients. Therefore, ERCC1 protein expression might be a useful biological indicator in patients with locally advanced NSCLC when planning clinical studies by testing the hypothesis of customized chemotherapy.

## Abbreviations

NSCLC: Non-small lung cancer; ERCC1: Excision repair cross-complementation group 1; CT: Computed tomography; OS: Overall survival; HR: Hazard ratio.

## Competing interests

The authors declare that they have no competing interests.

## Authors’ contributions

PW carried out the immunoassays. DY participated in the design of the study and performed the statistical analysis. WC and GA conceived of the study, and participated in its design and coordination; they also helped to draft the manuscript. All authors read and approved the final manuscript.
